# Association between having a primary care physician and health behavioral intention in Japan: results from a nationwide survey

**DOI:** 10.1186/s12875-023-02238-8

**Published:** 2023-12-19

**Authors:** Kemmyo Sugiyama, Takashi Oshio, Susumu Kuwahara, Hiromi Kimura

**Affiliations:** 1 Department of International and Community Oral Health, Tohoku University Graduate School of Dentistry, Sendai, Miyagi Japan; 2 Department of Community Health, Public Health Institute, Shiwa, Iwate Japan; 3 Institute of Economic Research, Hitotsubashi University, Kunitachi, Tokyo Japan; 4 Survey Research Center, Tokyo, Japan

**Keywords:** Health behavioral intention, *Kakaritsuke-I*, Primary care physician, Propensity score matching

## Abstract

**Introduction:**

Introducing a primary care physician (*Kakaritsuke-I*: KI) system to improve the efficiency of the health care system has been controversial in Japan. This study aimed to determine the relevance of KI to an individual’s health behavioral intentions.

**Methods:**

We used data from a nationwide, population-based internet survey (*N* = 5,234) to conduct a cross-sectional regression analysis. Additionally, we used a propensity score matching method to mitigate the potential endogenous biases inherent in the decision to have a KI.

**Results:**

KI was positively associated with various behavioral intentions. For example, the probabilities of intending to eat a well-balanced diet and engaging in moderate exercise were 12.8 (95% confidence interval [CI]:9.5–16.1) percentage points and 7.2 (95% CI: 3.9–10.4) percentage points higher, respectively, among those with a KI than among those without a KI. A KI equally increased the likelihood of getting vaccinated against coronavirus (in November 2021) by 7.5 (95% CI: 5.2–9.8) percentage points.

**Conclusions:**

Although further analysis is needed to examine the effect of KI on health, the results of this study suggest the potential benefits of policy measures to promote the KI system.

## Background

A *Kakaritsuke-I* (KI) has often been referred to as a key concept in Japan’s medical care system [1–5], although its institutional role is undefined [1, 2]. A KI is a concept close to what is called a family physician or family doctor in Western countries [1]. A KI often runs a community-based clinic with a small number of beds, makes a diagnosis, provides medical treatment, and gives a referral to the hospital to the patients when needed [1, 2, 4]. Under a free-access healthcare system and without a concept of family medicine, Japanese patients can freely choose a clinic or physician by themselves. A physician who has constructed a long-term relationship with a patient is generally considered a KI by a patient. Neither a physician nor the patient is required to establish a formal relationship. Hence, an individual’s perception of a KI is mainly subjective and likely affected by individual attributes such as age, socioeconomic status, health status, and health consciousness [6, 7].

A KI differs from a primary care physician in the US (who provides both the first contact for a person with an undiagnosed health concern as well as continuing care of various medical conditions) and a general practitioner in the UK (who authorizes access to specialty care, hospital care, and diagnostic tests) [1]. In addition, a KI does not need to be specialized in internal medicine, unlike a primary care physician or general practitioner.

In recent years, introducing a more formal KI system to improve the efficiency of the health care system has been controversial in Japan. Notably, the Japan Medical Association and Four Hospital Associations have been emphasizing a KI’s role. They define a KI as a locally based and reliable physician who has comprehensive capabilities in community health, public health, and welfare, is available for consultation on any health issue, has a good understanding of advanced healthcare information, and can refer patients to specialists or specialized healthcare facilities when needed [8].

In addition, recent discussions have revealed that implementing a KI system is critical for long-term medical care [9]. The COVID-19 pandemic has revealed that resources are limited for both hospital services and KI clinics. Besides such emergency situations, the complementary network of fundamental health care provided by KI and advanced medical care by hospitals is far more important for long-term medical care in a super-aged society.

However, little is known about the impact of KI on patients’ health behaviors. A study on *Kakaritsuke-Yakuzaihi*, the pharmacist version of KI, reported its beneficial effects were limited to a specific patient population [10]. Regarding KIs, one study reported that having a KI was positively associated with visiting large-scale hospitals [11]; however, it reflected the need for a practitioner referral for the initial hospital visit.

Thus, limited studies have investigated how a patient’s health behavior or intention is associated with having a KI. In addition, the fact that residents in Japan are not officially required to have a KI makes it possible to examine the impact of having a KI, unlike in Western countries where almost all residents are registered in the GP or GP-like system.

Herein, we examined the significance of KI for an individual’s health behavioral intention using data from a nationwide population-based internet survey. After conducting propensity score matching (PSM) [12] between respondents who perceived that they had a KI and those who did not, we examined how the perception of having a KI was associated with several aspects of health behavioral intention. Additionally, we examined the association between having a KI and receiving coronavirus vaccination, highlighting the importance of KIs during the coronavirus pandemic.

## Methods

### Study sample

This study used data from a population-based, nationwide internet survey conducted from late October to early November 2021, 1 month after the Coronavirus Disease 2019 (COVID-19)-related state of emergency was lifted by all prefectures on September 30, 2021. Registrants of an online survey company were included in this study. Approximately three-quarters of the registrants were distributed evenly between each prefecture, between men and women, and among five age groups (15–24, 25–34, 35–44, 45–59, and > 60 years). The remaining one-fourth of the registrants were distributed to each sex-age group in each prefecture in proportion to each prefecture’s actual population size. Therefore, the sample is not representative of the Japanese population. We planned to collect data from approximately 5,000 individuals and made questionnaires available to the registrants during the survey period, and we obtained data from 5,234 individuals who participated in the survey. This study was approved by Research Ethics Committee of Hitotsubashi University (reference no. 2021C010). All methods were carried out in accordance with the guidelines and regulations provided by the Committee.

### Measures

#### *Kakaritsuke-I* and health behavioral intention

The survey asked respondents to choose from the following options:1 = *I have a KI*, 2 = *I have a doctor who comes to my mind as a KI*, 3 = *I have no KI*, and 4 = *I do not know*. We developed a binary variable for having a KI by allocating 1 to respondents who chose 1 or 2 and 0 to others. We equally considered the case of a narrowly defined binary variable for KI by allocating 1 only to those who chose 1 and 0 to others in the same question. In addition to the questions about having a KI, the survey inquired if the respondents had regular doctor visits. We statistically analyzed all the respondents; however, we focused on respondents with regular doctor visits, considering the possibility that those respondents were less healthy and hence more likely to have a KI.

Regarding health behavioral intentions, the survey asked the respondents whether they (1) have well-balanced diet, (2) do moderate exercise, (3) get enough sleep, (4) do not smoke, (5) do not drink excessively, (6) do not build up stress, (7) participate in regular health checkups, (8) others (that respondents feel are health seeking behaviors), and (9) do not do anything. We developed binary variables for Items (1) to (9) by allocating 1 to respondents who answered yes and 0 to others. We considered whether the respondent had been vaccinated at least once against coronavirus by the survey time (from late October to early November 2021), based on their reported experience.

#### Variables to explain the probability of having a *Kakaritsuke-I*

We considered self-rated health as an indicator of general health conditions to explain the probability of KI in the PSM analysis [13, 14]. The survey inquired about the participants self-rated health on a 5-point Likert scale (1 = *good*, 2 = *somewhat good*, 3 = *average*, 4 = *somewhat poor*, and 5 = *poor*). We constructed five binary variables corresponding to each SRH score. We considered sex, age (29 years or below, 30–39, 40–49, 50–59 years, and 60 years or above), educational attainment (junior high school, high school, junior college, and college or above), job status (regular employee [including manager], non-regular employee, unemployed, out of labor force, and student), household income (low, moderate, high), marital status (married, unmarried), and family (living with family members, living alone) at the individual level by constructing binary variables for each category to conduct further analyses for each of these variables. We further considered the number of doctors per population at the prefecture level to gauge accessibility to medical services based on official statistics in 2020 [15]. We constructed binary variables for each of low, moderate, and high levels.

### Analytic strategy

As a descriptive analysis, we compared the prevalence of each health behavioral intention between respondents with and without a KI unadjusted for any other variable. For the regression analysis, we used the PSM method to mitigate endogeneity biases related to KI for the regression analysis. To this end, we initially computed the propensity scores by estimating a logistic regression model to explain the probability of having a KI based on a respondent’s self-rated health and the other abovementioned attributes. Subsequently, we used with a caliper width equal to 0.2 of the standard deviation of the logit of the propensity score to perform simple nearest-neighbor matching with one neighbor [16]. We matched each respondent with a KI with a respondent without a KI whose propensity score was closest to that of the respondent. Some respondents without a KI may have had two or more matching respondents, whereas others may have had no matches and were therefore excluded from the analysis. We counted the number of matches for each respondent without a KI and used it as the frequency weight to compute the average treatment effect (ATE) of having a KI on each health behavioral intention. As a robustness check, we used the narrowly defined binary variable for KI to compute ATE.

In all the statistical analyses, we considered both the entire sample of all respondents and the subgroup of those with periodic doctor visits. We set the significance level at 0.05, and used the Stata software package (Release 17; StataCorp, Texas, US) for all statistical analyses.

## Results

### Descriptive analysis

Table 1 compares the key features of the respondents with and without a KI. Of the 5,234 respondents in this study’s dataset, the proportion of those who had a KI or a doctor who came to mind as a KI was 51.5%. As expected, respondents with a KI visited doctors much more often than those without; 66.6% of respondents with a KI had periodic doctor visits, well above 28.8% of those without a KI. Table 1 shows that respondents with a KI were more likely to be married, older, women, having poor self-rated health, or living with their family members. Graduates from college had a lower proportion, and those from junior college had a higher proportion among respondents with a KI than among those without. The proportion of regular employees was lower, and the proportion of self-employed and unemployed individuals were higher proportion, among those with KI compared those without one. This somewhat counterintuitive finding may reflect higher time flexibility among self-employed and unemployed individuals to consult physicians and the often-unrecognized role of industrial physicians in the workplace as *de facto* KIs for regular employees. Additionally, we found that household income or the number of doctors per capita at the prefecture level was not associated to having KI.

**Table 1:** Key features of respondents with and without a *Kakaritsuke-I*

Attributes		Total	Do you have a *Kakaritsuke-I*?		
			Yes	No	*p*-value
Proportions (%) and chi-squared tests for their differences					
Periodic doctor visits		48.3	66.6	28.8	< 0.001
Sex	Female	50.5	53.5	47.4	< 0.001
Marital status	Married	50.9	57.0	44.5	< 0.001
Family	Living alone	19.7	15.2	24.6	< 0.001
Educational attainment^a^	Junior high school	2.5	2.1	2.8	0.090
	High school	39.9	41.0	38.6	0.083
	Junior college	11.5	13.2	9.6	< 0.001
	College or above	46.2	43.7	48.9	< 0.001
Job status^a^	Regular employee	41.9	35.7	48.5	< 0.001
	Non-regular employee	19.7	20.3	19.0	0.235
	Self-employment worker	6.7	7.7	5.6	0.002
	Unemployed	20.8	26.2	15.1	< 0.001
	Out of labor force	2.9	2.9	2.9	0.979
	Student	8.0	7.2	8.9	0.022
Sample means and Welch’s *t*-tests for their differences					
Self-related health	*M*	2.43	2.57	2.27	< 0.001
(range: 1 [good] − 5 [poor])	*SD*	(1.14)	(1.15)	(1.11)	
Age	*M*	44.2	48.5	39.6	< 0.001
(years)	*SD*	(17.0)	(17.7)	(15.0)	
Household income	*M*	6.18	6.38	5.97	0.057
(annual, million JPY)	*SD*	(7.83)	(8.06)	(7.58)	
Number of doctors	*M*	2.73	2.73	2.72	0.811
(per 1000 persons)	*SD*	(0.45)	(0.44)	(0.45)	
*N*		5,234	2,698	2,536	

^a^ Chi-squared tests rejected the null hypotheses of independence between educational attainment and *Kakaritsukei*-I and between job status and *Kakaritsukei*-I, both at *p* < .001

Table 2 compares health behavioral intention between respondents with and without a KI, unadjusted for any other factor, among all respondents (left part) and among respondents who visited a doctor periodically (right). As seen in this table, all items of favorable health behavioral intention and coronavirus vaccination were observed more often among respondents with a KI, both among all respondents and those with periodic doctor visits (except for “others” among those with periodic doctor visits). Consistently, “do not do anything” was negatively associated with having a KI. The table also shows that among respondents with periodic doctor visits there was a greater proportion of those with favorable health behavior intention in both groups (with and without a KI), compared to all individuals.

**Table 2:** Crude comparisons of prevalence (%) of health behavioral intention between respondents with and without a *Kakaritsuke-I*

Health behavior	All individuals				Individuals with periodic doctor visits			
	Total	Do you have a Kakaritsuke-I?			Total	Do you have a Kakaritsuke-I?		
		Yes	No	*p*-value^a^		Yes	No	*p*-value^a^
Have well-balanced diet	52.8	60.2	45.0	< 0.001	56.3	61.0	45.0	< 0.001
Do moderate exercise	36.8	40.5	32.8	< 0.001	40.5	42.4	35.6	0.001
Get enough sleep	44.3	49.7	38.6	< 0.001	47.7	50.6	40.6	< 0.001
Do not smoke	42.7	45.7	39.5	< 0.001	46.1	48.1	41.3	0.002
Do not drink excessively	34.3	37.1	31.3	< 0.001	37.5	39.5	32.7	0.001
Do not build up stress	32.2	37.7	26.4	< 0.001	36.3	39.2	29.1	< 0.001
Participate in periodic health checkups	29.5	38.2	20.3	< 0.001	36.2	40.8	24.9	< 0.001
Others	1.4	1.9	0.8	0.001	1.7	1.9	1.2	0.245
Do not do anything	17.5	12.9	22.5	< 0.001	12.9	17.8	10.9	< 0.001
Got vaccinated against coronavirus	85.3	90.0	80.4	< 0.001	88.6	90.9	83.0	< 0.001
*N*	5,234	2,698	2,536		2,529	1,798	731	

^a^ For the Chi-squared tests of the difference in prevalence between respondents with and without *Kakaritsuke-I*

### Regression analysis

**Fig. 1:**
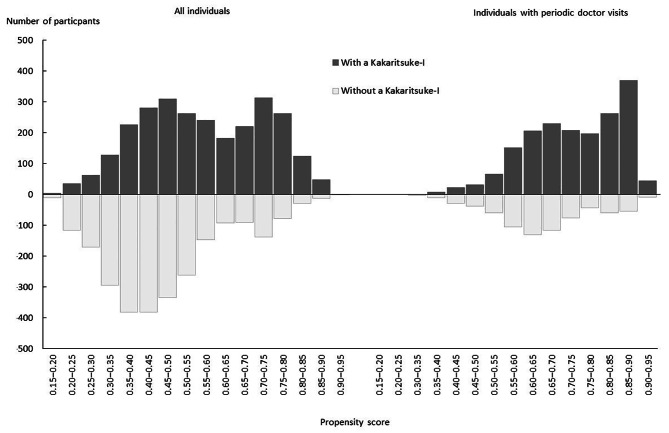
Comparison of propensity score histograms between respondents with and without a *Kakaritsuke-I* before matching

As the first step of the PSM method, Table 3 presents the estimation results of the logistic regression models that explain the probability of having a KI for all respondents (left) and respondents with periodic doctor visits (right). The baseline variables in each category correspond to the OR of 1 in this table. Low self-rated health, women, older age, and higher household income were positively associated with having a KI among all respondents, while living alone was negatively associated with it. Of these variables, only self-rated health and living alone continued to be associated with a KI among respondents with regular doctor visits. Figure 1 compares the histograms of the computed propensity scores between the respondents with and without a KI. The distribution of the propensity scores became less symmetric between the two groups, when the sample was limited to respondents with periodic doctor visits and the propensity scores were recomputed.

**Table 3:** Estimation results of logistic models to explain the probability of having a *Kakaritsuke-I*

Sample	All respondents		Respondents with periodic doctor visits	
Explanatory variables	OR^a^	95% CI^b^	OR	95% CI
Self-related health				
Good	1		1	
Somewhat good	1.14	(1.06, 1.24)	1.13	(0.99, 1.29)
Average	1.07	(1.01, 1.12)	1.14	(1.04, 1.24)
Somewhat poor	1.21	(1.15, 1.27)	1.16	(1.08, 1.25)
Poor	1.19	(1.11, 1.27)	1.15	(1.05, 1.26)
Sex				
Male	1		1	
Female	1.26	(1.11, 1.42)	1.07	(0.89, 1.30)
Age				
29 years or below	1		1	
30–39 years	0.90	(0.74, 1.09)	0.90	(0.67, 1.23)
40–49 years	1.23	(1.01, 1.50)	1.08	(0.80, 1.48)
50–59 years	1.70	(1.37, 2.12)	1.65	(1.17, 2.33)
60 years or above	3.90	(3.13, 4.85)	3.24	(2.30, 4.55)
Educational attainment				
Junior high school	1		1	
High school	1.22	(0.84, 1.78)	1.13	(0.62, 2.08)
Junior college	1.36	(0.90, 2.04)	1.21	(0.63, 2.33)
College or above	1.15	(0.79, 1.67)	1.12	(0.61, 2.05)
Job status				
Regular employee	1		1	
Non-regular employee	1.05	(0.89, 1.25)	0.86	(0.66, 1.12)
Self-employment worker	1.23	(0.96, 1.58)	1.38	(0.91, 2.10)
Unemployed	1.05	(0.86, 1.28)	1.05	(0.77, 1.42)
Out of labor force	1.12	(0.79, 1.58)	1.16	(0.67, 2.02)
Student	1.53	(1.19, 1.95)	0.96	(0.65, 1.42)
Household income				
Low				
Moderate	1.24	(1.07, 1.45)	1.19	(0.94, 1.51)
High	1.48	(1.24, 1.78)	1.22	(0.92, 1.62)
Marital status				
Unmarried	1		1	
Married	1.01	(0.87, 1.18)	0.99	(0.78, 1.25)
Family				
Living with family members	1		1	
Living alone	0.63	(0.53, 0.75)	0.59	(0.45, 0.77)
Number of doctors per population at the prefecture level				
Low	1		1	
Moderate	0.93	(0.81, 1.08)	0.92	(0.74, 1.15)
High	0.99	(0.86, 1.14)	1.10	(0.88, 1.37)
*N*	5,234		2,529	

^a^ Odds ratio

^b^ Confidence interval

Based on these results, Table 4 reports the ATE of having a KI on each health behavioral intention to examine the extent to which having a KI increases the probability of each health behavioral intention. As seen in this table, having a KI had a positive association with all types of health behavioral intentions except “others,” while it was negatively related to “do not do anything.” Specifically, the probabilities of intending to have a well-balanced diet and performing moderate exercise were 12.8 (95% confidence interval [CI]: 9.5–16.1) percentage points and 7.2 (95% CI: 3.9–10.4) percentage points higher, respectively, among those with a KI than among others. These effects were substantial in magnitude, judging by the prevalence of each health behavioral intention (see the figures in the column “Total” in Table 2). Furthermore, the probability of getting vaccinated against coronavirus (as of November 2021) also was 7.5 (95% CI: 5.2–9.8) percentage points higher with a KI. Similar results were obtained for individuals with periodic doctor visits. A closer examination of the results reveals that some types of intention (e.g., eating a well-balanced diet) had higher ATEs among those with periodic doctor visits, compared to all individuals. Meanwhile, other types (e.g., to participate in periodic health checkups) had lower ATEs in those with periodic doctor visits than for all individuals, and smoking status was not associated with a KI.

**Table 4:** Propensity-score-matching-based comparisons of health behavioral intention with and without a *Kakaritsuke-I*^a^

Health behavioral intention	All respondents		Respondents with periodic doctor visits	
	ATE^b^	95% CI^c^	ATE	95% CI
				*percentage points*
Have well-balanced diet	12.8	(9.5, 16.1)	15.4	(10.0, 20.9)
Do moderate exercise	7.2	(3.9, 10.4)	8.8	(3.3, 14.4)
Get enough sleep	9.5	(6.2, 12.8)	10.3	(5.2, 15.4)
Do not smoke	4.5	(1.3, 7.8)	4.0	(–1.6, 9.5)
Do not drink excessively	4.1	(0.9, 7.2)	6.2	(1.1, 11.4)
Do not build up stress	9.0	(5.8, 12.2)	12.4	(7.3, 17.5)
Participate in periodic health checkups	13.4	(10.5, 16.3)	12.1	(7.0, 17.2)
Others	0.4	(–0.5, 1.0)	0.7	(–0.6, 2.0)
Do not do anything	–0.9	(–1.1, –0.6)	–0.8	(–1.3, –0.4)
Got vaccinated against coronavirus	7.5	(5.2, 9.8)	6.5	(2.5, 10.5)
*N*	5,234		2,529	

^a^ Based on the estimation results of the logistic regression models reported in Table 3

^b^ Average treatment effect

^c^ Confidence interval

For the robustness test, we repeated the same analysis using a narrowly defined variable for having a KI and presented the results in Table 5. The results were largely in line with those shown in Table 4, although the estimated ATEs were moderately lower than those in Table 4. Smoking, excessive drinking, or physical exercise was not associated with KI.

**Table 5:** Propensity-score-matching-based comparisons of health behavioral intention with and without a *Kakaritsuke-I* using the narrowly definition of *Kakaritsuke-I*^a^

Health behavioral intention	All respondents		Respondents with periodic doctor visits	
	ATE	95% CI	ATE	95% CI
				*percentage points*
Have well-balanced diet	11.0	(7.6, 14.5)	13.4	(8.7, 18.1)
Do moderate exercise	4.7	(1.0, 8.3)	1.8	(–3.0, 6.6)
Get enough sleep	7.7	(3.9, 11.4)	7.4	(2.5, 12.3)
Do not smoke	1.0	(–2.9, 4.9)	1.6	(–3.4, 6.6)
Do not drink excessively	1.5	(–2.2, 5.3)	4.3	(–0.5, 9.1)
Do not build up stress	8.1	(4.2, 11.9)	7.4	(2.6, 12.1)
Participate in periodic health checkups	10.3	(6.9, 13.8)	5.3	(0.7, 9.9)
Others	1.4	(0.3, 2.4)	1.7	(0.1, 3.3)
Do not do anything	–0.6	(–0.9, –0.4)	–0.2	(–0.5, 0.1)
Got vaccinated against coronavirus	7.5	(5.0, 9.9)	5.5	(2.5, 8.4)
*N*	5,234		2,529	

^a^ When constructing a binary variable for having a KI, only the respondents who answered *I have* a KI were allocated one, and those who answered *I have a doctor who comes to my mind as a KI* were not considered having a KI.

^b^ Average treatment effect

^c^ Confidence interval

## Discussion

This study examined the association between KI and health behavioral intention using data from a nationwide, population-based internet survey. The proportion of those who had a KI or a doctor who came to mind as a KI was somewhat higher in our results than in an official survey conducted in 2019 (*N* = 3,000) [17] (51.5% vs. 45.0%; *p* < .001), although the latter survey did not ask the participants whether they had a doctor who came to mind as a KI. After employing the PSM method between respondents who had a KI and those who did not, we observed a close association between having a KI, and favorable health behavioral intentions and coronavirus vaccination.

These results remained largely intact even when we focused on respondents with regular doctor visits, highlighting the importance of the perception of having a KI for health behavioral intentions. However, the impact of having a KI increased for some intentions while decreasing for others, implying that the confounding effect of regular doctor visits on the impact of having a KI on health behavioral intention may not be uniform across types of intention.

We confirmed the impact of having a KI on health behavior intention, even if we used a narrowly defined variable for having a KI and focused on whether the respondent reported having a KI. However, with this narrow estimation, the estimated impact of having a KI moderately diminished, suggesting that having a close relationship with a physician, even if he/she is not specifically defined as a KI, may generally affect health behavior.

Based on a previously described definition [8], KI generally relied on daily medical care. Due to a lack of data, our study did not focus on health outcomes, including mortality or the incidence of specific diseases, dementia, or disability. However, studies in other countries have rarely investigated the impact of GP on health outcomes. Instead, we focused on health behaviors and other important factors that a KI should discuss with patients in preventive care [8].

KI had a positive effect on the propagation of COVID-19 vaccination, according to our results. Historically, primary care physicians have played a vital role in vaccinations among the general population [18], and this has been widely true for COVID-19 vaccination [19]. Our results are consistent with previous studies. Furthermore, the high vaccination rate observed in this study was most likely influenced by the fact that it was conducted during the state of emergency declaration of COVID-19. The effects of increasing telemedicine use during this period should be considered. In 2018, the ban on telemedicine was officially lifted [20]. Therefore, the general population who did not previously have a KI may have had more opportunities to consult a physician regularly after their first visit using telemedicine. Further studies are needed to compare the proportion of KI patients before and after the COVID-19 pandemic.

This study has several limitations. First, the definition of KI was based on respondents’ perceptions of having a KI, and the reported health behavioral intention was based on participants’ subjective assessment. Both suggest the possibility of non-differential misclassification, which may imply that the resultant odds ratio was biased towards the null and hence limit the reliability of the estimation results. Second, because our study was cross-sectional, we cannot completely rule out causality, although we used PSM to address endogeneity and simultaneity. Participants who were more concerned about their health may have visited doctors more frequently. Furthermore, in addition to a lack of formal assessment about the validity of the survey, we recognize potential selection biases inherent in an Internet survey – such as biases towards young people, frequent Internet users, and urban residents which may imply higher health literacy and/or easier access to health care services and hence lead to an overestimated probability of having a KI. We should also consider the problems due to the small sample size (5,234 respondents) and limited generalizability of the estimation results (reflecting lack of representativeness of the Japanese population and the timing of the survey [conducted during the COVID-19 pandemic]).

## Conclusions

Although further analysis is needed to examine the effect of KI on health, the results of this study suggest the potential benefits of policy measures to promote the KI system. Longitudinal data and follow-up studies are required to track the evolution of actual health behavior and outcomes over time to precisely capture the impact of having a KI on the population’s health. A more specific and clearer institutionalization of KIs is required to realize their potential benefits.

## Data Availability

The datasets used and/or analyzed during the current study are available from the corresponding author on reasonable request.

## List of Abbreviations

ATE: Average treatment effect
CI: Confidence interval
COVID-19: Coronavirus Disease 2019
GP: General practitioner
KI: *Kakaritsuke-I*
PSM: Propensity score matching
